# Current management of eosinophilic granulomatosis with polyangiitis across Europe: insights from a multinational expert survey

**DOI:** 10.1093/rheumatology/keag218

**Published:** 2026-04-21

**Authors:** Edoardo Conticini, Giacomo Emmi, Silvia Grazzini, Marc André, Jan Willem Cohen Tervaert, Eugenia Durante, Francesca Torracca, Aladdin J Mohammad, Frank Moosig, Thomas Neumann, Roser Solans Laque, Benjamin Terrier, Augusto Vaglio, Paolo Cameli, the European EGPA Study Group (EESG), Diego Bagnasco, Francesco Benvenuti, Alvise Berti, Milena Bond, Marco Caminati, Giulia Cassone, Francesco Cianci, Laura Coladonato, Fabrizio Conti, Christian Dejaco, Stefano Del Giacco, Paolo Delvino, Francesco Ferro, Marco Folci, Alberto Lo Gullo, Giuseppe Guida, Mario Malerba, Riccardo Mancini, Alessandra Milanesi, Sara Monti, Gianluca Moroncini, Luca Moroni, Santi Nolasco, Silvia Noviello, Paola Parronchi, Roberto Padoan, Stefano Pizzimenti Hissaria Pravin, Beatrice Ragnoli, Francesca Regola, Luisa Ricciardi, Barbara Ruaro, Lorenzo Salvati, Jan Walter Volk Schroeder, Savino Sciascia, Marco Sebastiani, Paola Tomietto

**Affiliations:** Rheumatology Unit, Department of Medicine, Surgery and Neurosciences, University of Siena, Siena, Italy; Department of Medical, Surgical and Health Sciences, University of Trieste, Trieste, Italy; Clinical Medicine and Rheumatology Unit, Cattinara University Hospital, Trieste, Italy; Rheumatology Unit, Department of Medicine, Surgery and Neurosciences, University of Siena, Siena, Italy; Internal Medicine, G. Montpied Hospital, Clermont-Ferrand, France; Department of Medicine, Division of Rheumatology, University of Alberta, Edmonton, AB, Canada; Mental Health and Neuroscience Research Institute, Maastricht University, Maastricht, The Netherlands; APACS, Associazione Pazienti con Sindrome di Churg Strauss, Arosio, Italy; APACS, Associazione Pazienti con Sindrome di Churg Strauss, Arosio, Italy; Rheumatology, Department of Clinical Sciences, Lund University, Sweden, Lund; Department of Medicine, University of Cambridge, Cambridge, UK; Rheumazentrum Schleswig-Holstein Mitte, Universitätsklinikum Schleswig - Holstein, Neumünster/Kiel, Germany; Department of Rheumatology, Cantonal Hospital St. Gallen, St. Gallen, Switzerland; Internal Medicine, Hospital Vall Hebron, Barcelona, Spain; Department of Internal Medicine, National Referral Center for Rare Systemic Autoimmune and Autoinflammatory Diseases of Ile de France, East and West, Cochin Hospital, Assistance Publique-Hôpitaux de Paris, Paris, France; Université de Paris Cité, INSERM, U970, Paris-Centre de Recherche Cardiovasculaire, Paris Cité University, Paris, France; Nephrology and Dialysis Unit, Meyer Children’s Hospital IRCCS, Firenze, Italy; Department of Biomedical, Experimental and Clinical Sciences, University of Firenze, Firenze, Italy; Respiratory Diseases Unit, Department of Medicine, Surgery and Neurosciences, University of Siena, Siena, Italy

**Keywords:** eosinophilic granulomatosis with polyangiitis, vasculitis, education (patients), attitude of health professionals, outcome measures, biological therapies, comorbidity/multimorbidity

## Abstract

**Objectives:**

Real-world practice patterns of eosinophilic granulomatosis with polyangiitis (EGPA) remain poorly defined. This study aimed to describe current diagnostic and therapeutic approaches across experienced European centres, identifying areas of convergence and variability to inform future standardization of care.

**Methods:**

We distributed a 44-item online survey covering diagnostic evaluation, treatment strategies, patient-reported outcome measures (PROMs) and the role of patient advocacy groups. The survey was reviewed by an expert panel and disseminated within the European EGPA Study Group. Responses were collected anonymously between April and August 2025 for statistical analysis.

**Results:**

Fifty-four experts from six countries participated, most with long-standing experience and substantial EGPA caseloads. Multidisciplinary care and screening for cardiac and renal involvement were widely adopted; histological confirmation was reported in fewer than 25% of cases. Treatment strategies varied considerably: over half of respondents initiated anti-IL-5 therapy at diagnosis, and the combination of glucocorticoids, rituximab and mepolizumab was the preferred induction regimen in severe disease. CS tapering protocols differed, with most clinicians targeting withdrawal within 12 months. PROMs and disease-specific questionnaires were used inconsistently, despite broad recognition of their value. Advocacy groups were viewed as crucial, particularly for patient education and referral.

**Conclusion:**

This first multinational survey reveals substantial heterogeneity in real-world diagnostic and therapeutic practice, reflecting gaps in validated criteria, standardized activity measures and treatment algorithms. These findings highlight the need for coordinated prospective research and harmonized evidence-based guidance to optimize outcomes for patients with EGPA.

Rheumatology key messages

- EGPA diagnosis is heterogeneous due to lacking validated criteria and disease-specific assessment tools.
- Early anti-IL5 use and triple induction therapy are frequently adopted in clinical practice.
- Harmonized management strategies and prospective collaborative studies are urgently needed to optimize outcomes.

## Introduction

Eosinophilic granulomatosis with polyangiitis (EGPA) is a small vessels vasculitis belonging to the subgroup of ANCA-associated vasculitis (AAV).

Once underdiagnosed and somewhat excluded by randomized clinical trials among AAV due to its rarity and the substantial lack of any effective drugs, the recent approval of two monoclonal antibodies, as well an increasing awareness of the disease, a growing interest in terms of research and the launch of several trials, have shed a new light in the comprehension and the effective management of this condition.

EGPA remains the rarest of AAV and its incidence, apparently stable during the last decades, ranges from 0.1–4/1 000 000 in Europe; nevertheless, the prevalence is strikingly raising (from 14 to 36.6/100 000 in Southern Sweden) [1], suggesting the increase in awareness of the disease plus a better and more effective approach.

Such a rarity, probably be associated with misdiagnosis and underdiagnosis, therefore leading to underestimate the magnitude of the disease.

Furthermore, aside from the real number of patients affected by EGPA, it is undisputable that centres actively involved in the overall management and research remain too few and several shortcomings still hamper the effective treatment of this condition: first, EGPA is a protean disease and patient can be variously referred to different specialist whose therapeutic and diagnostic approach may differ. Secondly, no diagnostic criteria have been validated nor any specific disease activity score has ever been proposed, while the most employed ones (such as Birmingham Vasculitis Activity Score (BVAS)) may overlook certain aspects of the disease and, on the opposite, point on symptoms and signs usually absent in this disease. Third, which is a consequence of the previous point, organ specific questionnaires and scores have been adopted from other conditions [2]. Fourth, in terms of treatment, uncertainty exists about the optimal length and dosage of immunosuppressants and glucocorticoids (GCs): recommendations themselves refrain from giving clear indications about the tapering of oral GCs, which is usually adapted from other vasculitis [3]. Lastly, real-life patients may differ from RCTs ones, both in terms of inclusion criteria and clinical features.

Therefore, goal of this study was to provide a pictorial overview of how European experts currently diagnose and treat EGPA in their daily clinical practice, aiming to eventually align therapeutic and diagnostic procedures across European centres and to provide a ground for further research.

## Methods

Within the frame of European EGPA Study Group (EESG) (official site: https://eestudygroup.com), we designed an online survey, with the participation of specialists in rheumatology, allergology-immunology, pulmonology, nephrology, internal medicine and otolaryngology active throughout Europe with a specific expertise in the management of EGPA. A board of nine experts, all from different countries, was individuated and revised the first draft of the survey, which was presented during the ninth meeting of EESG on 23 May 2025, in Trieste, Italy, and underwent a second round of revision before the dissemination to all members of EESG.

The survey, which was administered via Google forms, consisted of a total of 44 questions, subdivided in five macro-areas: general questions, diagnostic dilemmas (scenario of a patient without a previous diagnosis of EGPA), the challenge of treatment, patient-reported outcome measures (PROMs) and the role of patients’ advocacy organizations (Supplementary Data S1). The questions were mainly single-choice format, but multiple choices format was also used.

The opinions were anonymously collected from April to August 2025, while preliminary results were discussed during the 10th meeting of EESG on 18 September 2025, in Paris, France.

### Statistical analysis

For statistical purposes, therapeutic approaches were dichotomized (i.e. induction treatment coded as 0 for single-agent therapy and 1 for combination therapy). The frequencies of the variables were compared across cohorts using contingency tables, and statistical significance was assessed with the *χ*^2^ test or Fisher’s exact test, as appropriate. A *P* < 0.05 was considered significant.

## Results

Fifty-four experts and specialists (31 males, 57.4%) from Italy (47 participants, 87%), Serbia (1, 1.8%), Portugal (1, 1.8%), UK (2, 3.7%), France (2, 3.7%) and Czech Republic (1, 1.8%) participated in the survey. More than half had at least 10 years of practice and up to 59.2% had no <30 EGPA patients in follow-up. Regarding the age of participants, the most represented was the fourth decade (53.7% aged 31–40, followed by 24.1% being 41–50) while, in terms of specialization, the two most represented were rheumatology (20, 37%) and immuno/allergology (19, 35.2%) (Supplementary Fig. S1).

### Diagnostic dilemmas

**Figure 1:**
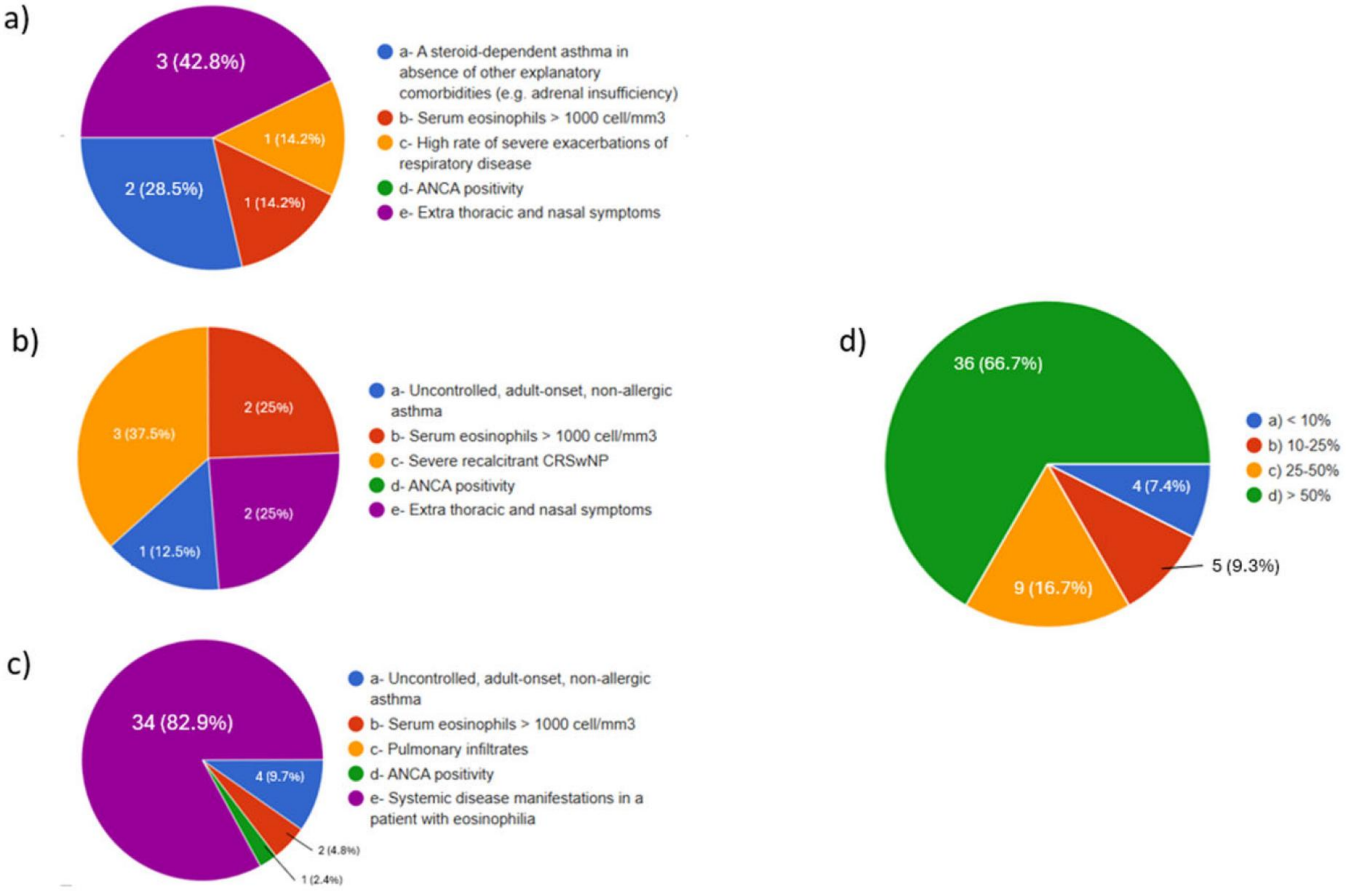
On the left, responders were asked to choose the most important red flag of EGPA: results are stratified according to the specialization: pulmonologists (**a**), otolaryngologists (**b**) and rheumatologists, immunologists and internal medicine specialists (**c**). On the right, participants were requested to declare the percentage of patients with EGPA managed with at least another specialist

Despite such a scattering distribution, mirrored by what is considered the most important red flag of EGPA (extra-thoracic and nasal symptoms for 42.8% of pulmonologists; severe recalcitrant CRSwNP for 37.5% of otolaryngologists; systemic disease manifestations in a patient with eosinophilia for 82.2% for rheumatologists, immunologists and internal medicine specialists), most of our responders declared to manage >50% of their patients in collaboration with at least one another specialist (Fig. 1).

In line with currently available guidelines, more than 80% of experts recommend ECG and echocardiography and ask for serum creatinine, routine analysis and protein/creatinine ratio also in patients without any sign or symptom of cardiac and/or renal involvement. Remarkably, in asymptomatic patients, 35.2% also ask for troponin and 14.8% for cardiac MRI, while in case of suspected heart involvement, the endomyocardial biopsy (EMB) is warranted by 31.5% of participants (Supplementary Fig. S2).

Nevertheless, even though a histological confirmation is considered important in cases of suspected glomerulonephritis, skin vasculitis and enteritis by 83.3%, 72.2% and 63% of responders, respectively, the availability of histological feature of granulomatous inflammation or vasculitis is usually available in <25% of cases, according to the opinion of 68.5% and 75.9% of the experts, respectively.

### Treatment

When asking about the preferred therapeutic approach, both in severe and non-severe EGPA, 53.7% of physicians tend to prescribe anti-IL5 agents immediately at diagnosis, while in 18.5–29.6% of severe and non-severe, respectively, in case of relapse.

**Figure 2:**
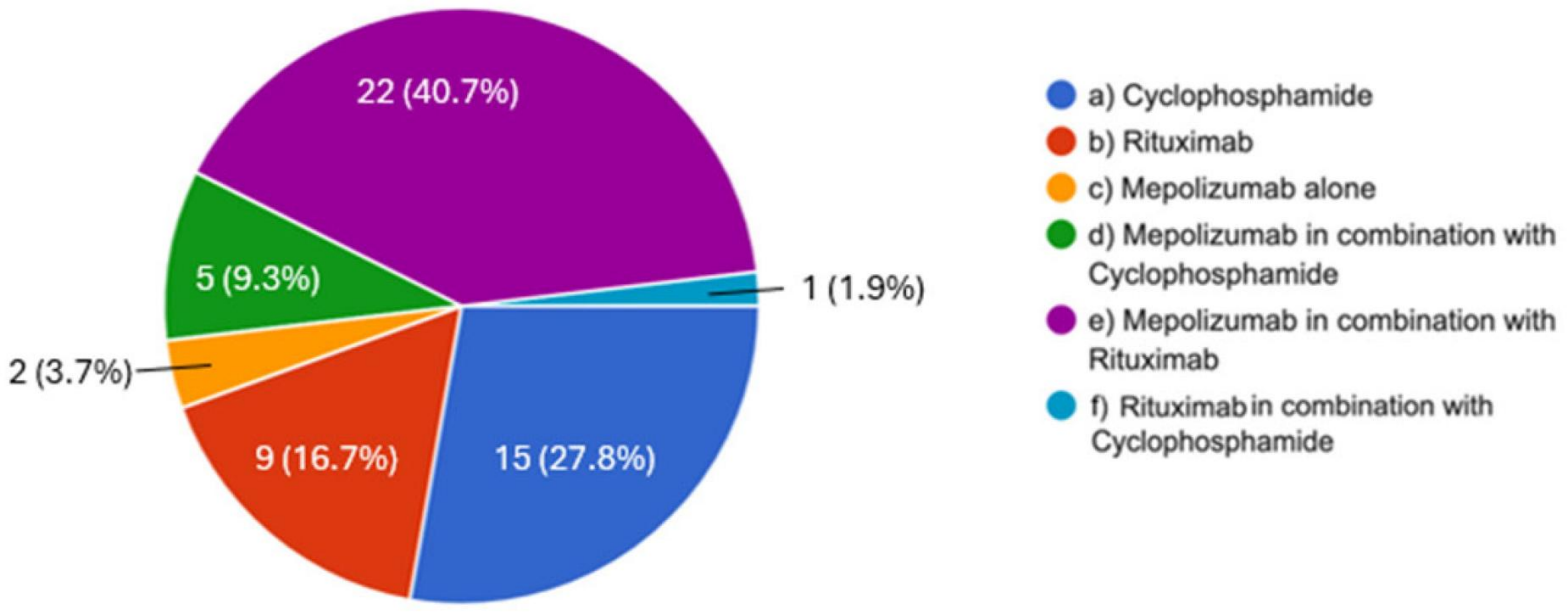
Preferred therapeutic approach of induction in organ and/or life-threatening EGPA. All options are intended in combination with systemic glucocorticoids

Specifically, focusing on organ or life-threatening vasculitis, the triple therapy of GCs + mepolizumab (MEPO) + rituximab (RTX) is the preferred option (40.7%), followed by CYC (27.8%) and RTX (16.7%) alone (Fig. 2). Those who answered to use a combination of RTX + MEPO as induction treatment were asked to specify the time of administration of MEPO, distinguishing between ‘true’ combination (i.e. MEPO was started within 3 months from the last infusion of RTX) and sequential therapy (i.e. MEPO was started at least 3 months after the last infusion of RTX): 54.2% start MEPO in combination with RTX, while 16.7% opt for a sequential treatment and 29.2% variously employ both options.

Still on EGPA, two-third of responders start with standard dosage of 300 mg/4 weeks, while 25.9% choose according to signs and symptoms.

The positioning of benralizumab is similar to MEPO, being considered as an equivalent alternative for 57.4%, while roughly 40% place it as a second-line treatment.

For remission maintenance in patients treated with RTX at induction, anti-CD20 is administered for 12–24 months by 50% of experts, while 38.9% opt for a longer period and 11.1% do not use it except for induction of remission.

**Figure 3:**
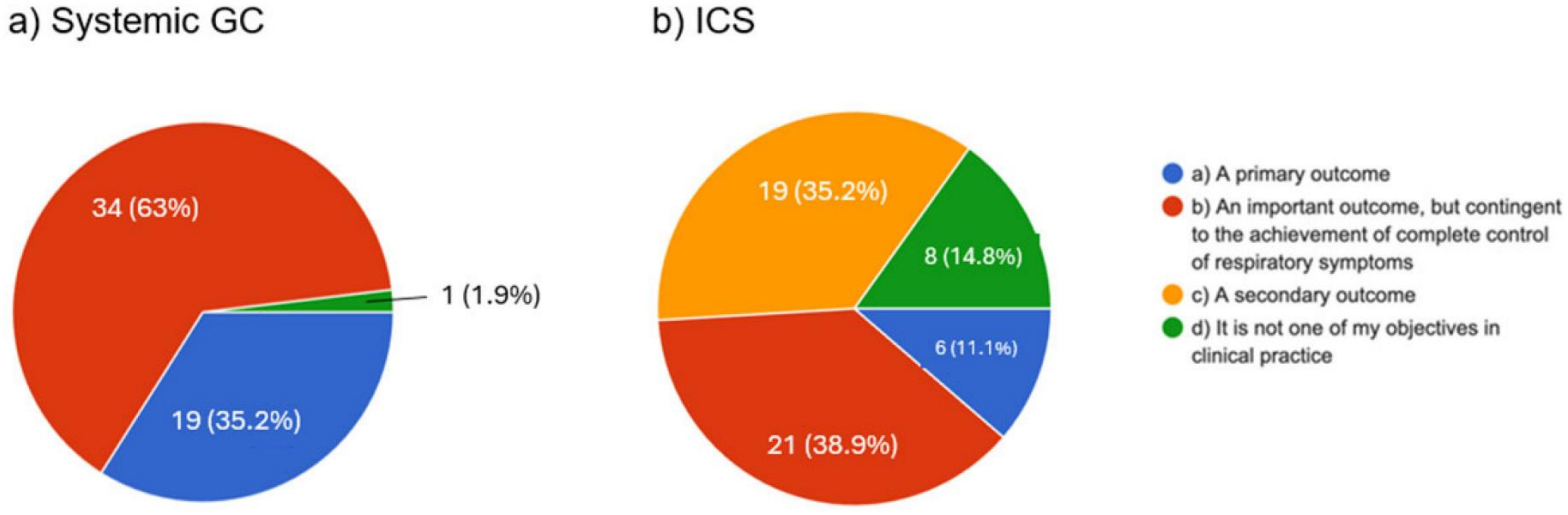
Attitude of EGPA specialists regarding permanent systemic steroids discontinuation (**a**) and ICS sparing (**b**). Abbreviations: ICS: inhaled corticosteroids, GC: glucocorticoids

Specifically focusing on GCs, complete remission with no OCS can be achievable in most patients, according to the vast majority of responders, and represents a primary outcome in two-third of experts, differently from inhaled CSs (ICS), whose discontinuation is seen mainly as a secondary outcome (Fig. 3). Consequently, a complete discontinuation of GCs within 12 months is suggested by 55.6% of responders, while <10% prescribe a chronic, low dosage of steroids. If asked about MEPO administration in patients in full remission still taking daily low dosage of GCs, 90.7% those who start with 100 mg/4 weeks upscale to 300 mg, while, on the opposite, a tapering of anti-IL5 agents is considered by up to 75% of responders: in particular, 14.8% consider a downscale to 100 mg/4 weeks in the majority of patients, 48.1% in selected cases and 11.3% only in case of adverse events (Supplementary Fig. S3).

Concerning respiratory outcomes, regardless of the specialization, the improvement of pulmonary functional parameters is considered a primary outcome by most of our responders and a regular follow-up (at least twice a year) is recommended in 59.3% of patients (Supplementary Fig. S4).

### PROMs

**Figure 4:**
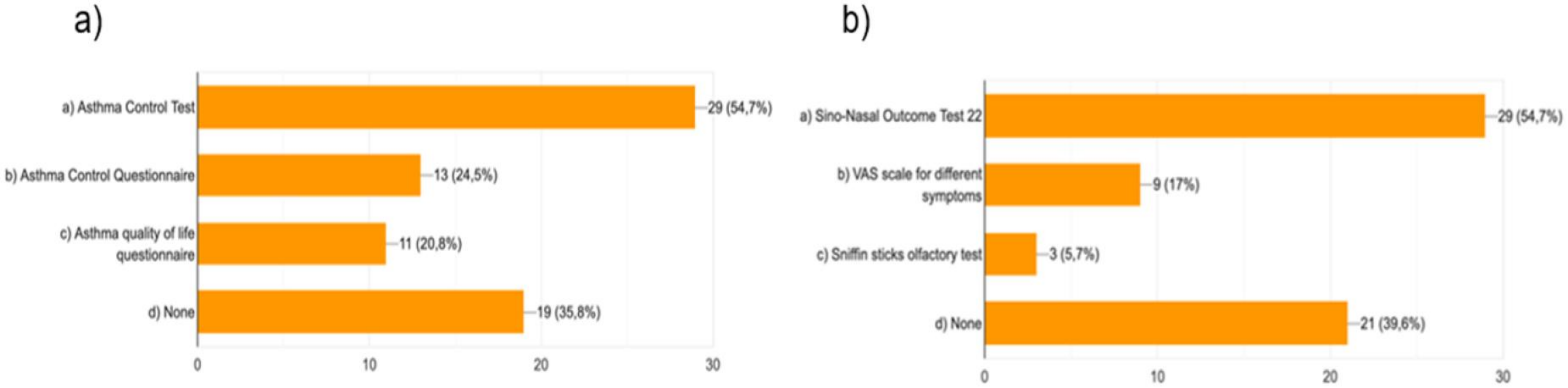
Frequency of implementation in the routinary practice of questionnaires concerning the clinical control and symptoms burden of asthma (**a**) and CRSwNP (**b**). Abbreviations: CRSwNP: chronic rhinosinusitis with nasal polyposis

Regarding the goal of the clinical practice, the improvement of the quality of life is the most valuable objective for their patients in 46.3% of responders, but the use of specific questionnaires to evaluate such an end point is uncommon, being performed in <50% of patients by roughly three-fourths of experts (Supplementary Fig. S5). The use of questionnaires is uncommon also for assessing asthma control and ENT symptoms, being performed in no >60% of cases (Fig. 4).

### The role of patients’ advocacy organization

**Figure 5:**
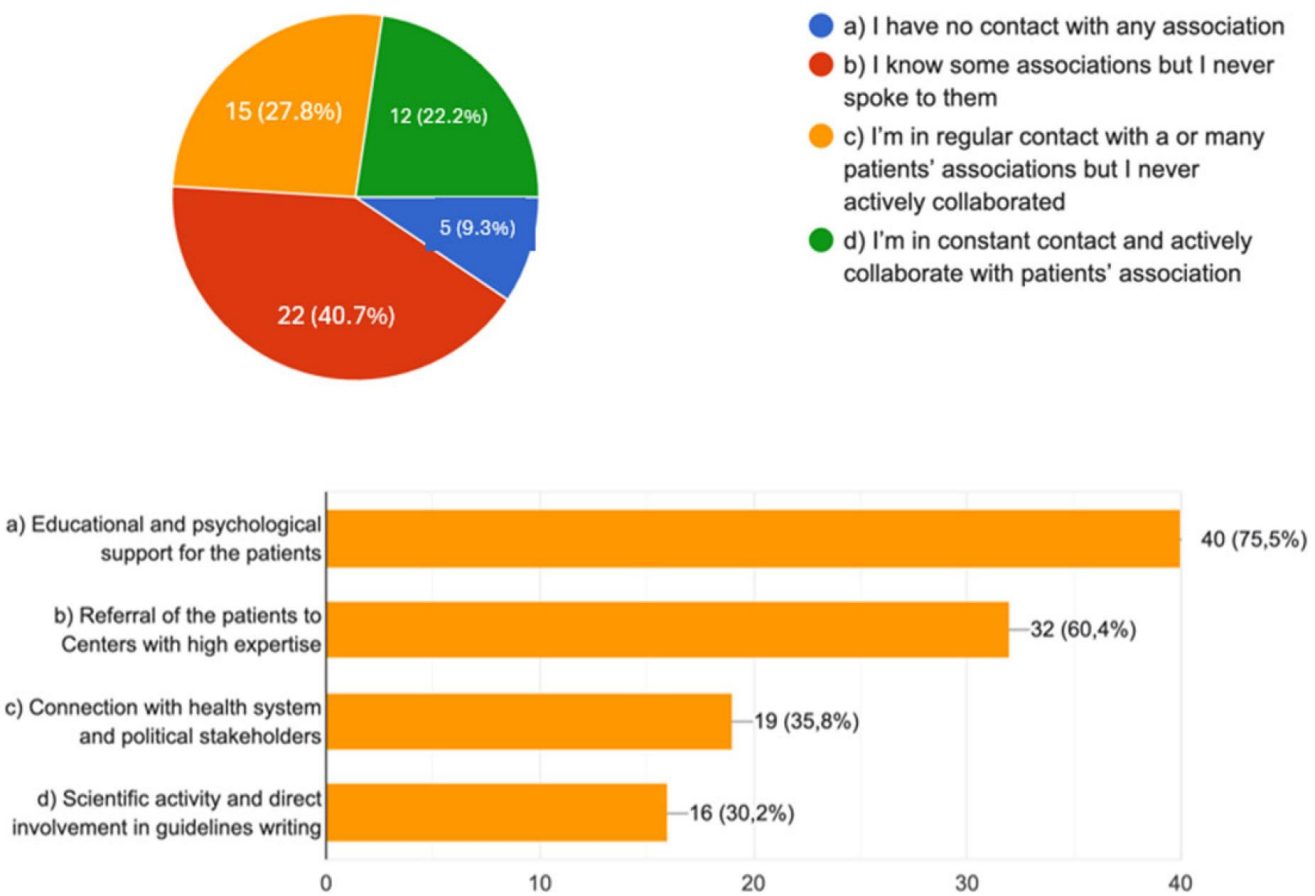
Participants were asked to state their current relationship and collaboration with EGPA patient association (upper image) and to declare what they believe should be the primary goals of such association (lower image)

Finally, the importance of patients’ association is strongly recognized by most physicians, being >50% in contact with them and remarking the role in terms of educational support to the patients, which is considered the most important activity by 75.5% of participants. Thirty-two respondents (60.4%) believed that patients associations are also fundamental for a proper referral of patients to referral centres, while connection with health systems and political stakeholders as well as collaboration in scientific activity are considered prominent only for 35.8% and 30.2% of experts, respectively (Fig. 5).

**Figure 6:**
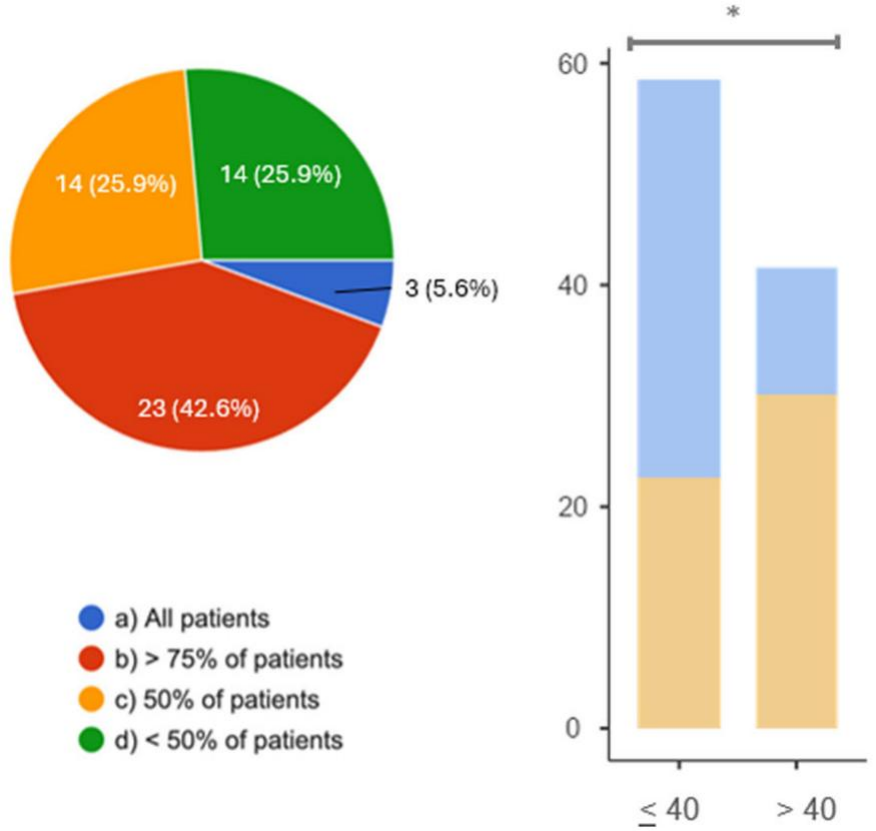
Clinicians expectations about the achievement of a complete clinical remission of disease (defined as BVAS = 0) with no OCS at diagnosis of EGPA. On the right, survey participants were stratified according their age. Turquoise subcolumn comprises the answers ‘all patients’ and ‘> 75% of patients’. *: *P* = 0.015

Further sub-analysis was performed to explore any correlation between responders (in terms of age, years of practice, specialization and number of EGPA patients in follow-up) and answers. The only statistically significant differences were observed concerning treatment approach and outcomes prediction. Indeed, responders under 40 years were significantly (*P* = 0.015) more prone to believe that >75% of patients can achieve full, steroid-free, remission, while no difference was evidenced for any other variable (Fig. 6).

Additionally, 64% of those who believe in the possibility of the full remission, irrespective of age, specialization and years of practice, are more prone to prescribe triple therapy (GC + MEPO + RTX) for induction of remission in severe EGPA; moreover, 54.2% of them also tend to rapidly withdraw oral GCs, while, in the overall cohort of responders, the preferred tapering is within 12 months. Finally, a slightly difference was assessed in terms of administration of anti-IL5 agents, which are prescribed immediately at diagnosis of severe EGPA in 58.1% of physicians younger than 40, while 54.5% of those older than 40 in case of relapse or failure.

## Discussion

Our survey is the first one ever conducted aiming to provide a pictorial depiction of the state of art of EGPA in the real life.

In order to exclude diagnostic and therapeutic behaviours attributable to inadequate expertise or malpractice, the survey was conducted within a highly skilled context, being spread among those physicians who are members of an international disease-dedicated study group.

In particular, the medium number of patients treated by any physician allows to consider responders true ‘experts’, reasonably encompassing those European centres involved in most clinical trials and aware of currently available guidelines [4] and therapeutic novelties.

In this regard, despite a non-neglectable heterogeneity in terms of age, years of practice and specialization, we found a good adherence to diagnostic guidelines: most patients are seen in a multidisciplinary manner and a thorough screening of subclinical renal and cardiological involvement is carried out in the vast majority of subjects without an overt organ damage.

On the opposite, the availability of histological findings consistent with vasculitis and/or granulomatous inflammation remains an open issue, being available in a little minority of patients. Such an aspect could appear to be a contradiction, as all the many sets of criteria which have been proposed in the last 40 years [3, 5–8] focus on histological findings leading to objective evidence of vasculitis. Nevertheless, it should be remarked that none of them are diagnostic criteria, therefore in the clinical practice highly suggesting features of EGPA may make biopsy useful but not essential. Moreover, aside from kidney and skin, for which histology still has a paramount role, biopsies of sino-nasal district have a low sensitivity [9]; nevertheless, the rate of experts who positively answered to the question about biopsies for suspected nasal involvement remains lower than expected and may have been influenced by the reduced numerosity of otolaryngologists in our cohort. On the opposite, the rate of responders who find important EMB is relatively high, despite the recently published American College of Cardiology Consensus [10] and the modified Lake Louise criteria, evidencing an excellent negative predictive value of MRI [11].

When asking about the treatment, responses were curiously not fully in line with currently available recommendations and guidelines.

In particular, an early administration of anti-IL5 agents seems to be the preferred option for most responders. This would not be surprising for non-severe EGPA: the concept of ‘relapsing patients’ seems to be redundant, given the fact that EGPA relapses after GCs discontinuation or tapering are very common [12]. On the opposite, it is remarkable that the same rate of responders who administer MEPO at diagnosis (52%) is shared for both severe and non-severe forms: more in details, the combination of CYC or RTX + MEPO is the preferred approach for induction treatment in more than half of experts involved in this survey, being RTX the most employed immunosuppressant.

Although MIRRA trial included patients with non-organ or life-threatening vasculitis [8], a growing number of retrospective studies seems to prove the efficacy and safety of MEPO also in those with unfavourable prognostic factor [13]. At the same time, in light of the results of REOVAS trial and other real-world studies [14–16], RTX seems not inferior to CYC for induction of remission, being burdened by a similar adverse events risk.

Following these premises, the ‘triple therapy’ GCs + MEPO + RTX, to be simultaneously or sequentially administered, seems a reasonable approach and, although not investigated, could pave the way to further studies in order to achieve a faster remission in severe EGPA.

Another hot topic is the dosage of MEPO: although officially licenced at 300 mg/4 weeks, some studies have evidenced a substantial efficacy also of the lower dosage [17–19]. In line with this, one quarter of respond adopt a tailored approach, choosing the dosage according to the severity of symptoms.

Specularly, and maybe more surprisingly, the downscale of MEPO from 300 mg to 100 mg is a therapeutic approach endorsed by a non-neglectable rate of experts. Preliminary, interesting findings [20], although impaired by the reduced observational period and by the retrospective design, somehow reassure about the risk of flares: nevertheless, at the moment, caution should be exerted when tapering MEPO, given the high relapse risk and the substantial safety of the drug also at full dosage.

Analogously, specifically focusing on the use of immunosuppressant for remission maintenance in severe EGPA, approach may deeply vary among experts. In particular, the optimal length and dosage of RTX is far from being clearly defined, ranging from 12 to >36 months; following these premises, and also in light of the substantial lack of studies aiming to answer this question [16, 21, 22], the choose of balancing between infective and relapse risk relies only on clinician’s judgement, which is also hampered by the absence of reliable biomarkers other than eosinophils.

Such an uncertainty is even more striking in oral GCs discontinuation: despite considered an important outcome, to be reasonably achieved in most patients, more than half of physicians chooses a cautious protocol, halting OCs not before 12 months after induction. The ratio of this choice is uncertain, giving the fact higher than <7.5 mg of prednisone after 6 months from induction is associated with a significantly higher risk of infection and a higher mortality [23].

Unsurprisingly, inhaled CS sparing appeared not to be a burning issue for the participants, even though long-term exposure to moderate-to-high daily ICS is associated with a significant burden of steroid-related side effects [24]. This finding could be explained by the aforementioned persistent uncertainty for systemic GCs management and for definition of disease remission, which may lead the management of ICS to be underestimated in terms of priority; moreover, the relatively low percentage of pulmonologists among the participants may be a factor. Still, despite this aspect, the monitoring of pulmonary function test is one of the most important outcome for all physicians, regardless of their specialization, and periodic (at least once a year) assessment is asked for the totality of patients. Nevertheless, the use of disease-specific questionnaire is still overlooked in the clinical practice: Asthma Control Test and Sino-Nasal Outcome Test 22 are the most employed, but the rate of their use is slightly over 50%, while up to 40% of physicians do not use any kind of questionnaires. Given the effect of anti-IL5 agents also in the quality of life [2], the use of disease-specific questionnaire, preferably focused on EGPA only and not borrowed from any other conditions, would be a supportive tool for patients and physicians, too.

In conclusion, the present study provides a comprehensive depiction of current diagnostic and therapeutic practices in EGPA across European referral centres. Despite notable advancements, particularly a growing emphasis on multidisciplinary care, we observed a reliable variability in clinical decision-making, especially regarding treatment duration, CS tapering and implementation of disease activity tools and PROMs in the routinary practice. These discrepancies probably reflect the absence of validated diagnostic criteria and the need for disease-specific activity scores, as well as harmonized treatment algorithms tailored to the heterogeneity of EGPA. In this regard, national prescribing regulations and reimbursement policies may have influenced treatment selection, timing and duration, therefore shaping real-world therapeutic strategies independently of clinical reasoning. In addition, specialty-specific perspectives may have contributed to variability in diagnostic and therapeutic choices. The heterogeneous distribution of specialties within the cohort, while reflecting real-world care pathways, may have influenced the prioritization of organ involvement, diagnostic tools and treatment goals, further emphasizing the importance of multidisciplinary management in EGPA.

Nonetheless, the high level of engagement among experienced clinicians and the shared recognition of key clinical priorities suggest a readiness within the community to converge towards more standardized, evidence-based approaches. Our findings underscore the urgent need for prospective, collaborative studies to fill existing knowledge and aligning real-world practice with emerging evidence will be essential to optimizing outcomes and quality of life for patients living with EGPA.

## Supplementary Material

keag218_Supplementary_Data

## Data Availability

Data available on request.
