# Supplementary material for: Current management of eosinophilic granulomatosis with polyangiitis across Europe: insights from a multinational expert survey
Source: Rheumatology (Oxford). 2026 Apr 21;65(5):keag218. doi: 10.1093/rheumatology/keag218 (PMC13152655; doi:10.1093/rheumatology/keag218)
Supplement: keag218_Supplementary_Data [file keag218_supplementary_data.zip › rhe-25-3203-File002.pdf]

# A survey to better understand the different approaches in diagnosing and treating EGPA

## Macroarea 1: General questions

1. Sex
   Single-choice answer

   - Male
   - Female
   - Not binary
   - Prefer not to answer

2. Age
   Single-choice answer

   - a) <30
   - b) 31-40
   - c) 41-50
   - d) 51-60
   - e) 61-70
   - f) >70

3. Years of practice
Single-choice answer

- a) <5
- b) 5-10
- c) 10-20
- d) >20

4. Main specialization
Single-choice answer

- a) Rheumatology
- b) Respiratory Diseases
- c) Nephrology
- d) Internal medicine
- e) Immunology/allergology
- f) Cardiology
- g) Otorhinolaryngology
- Other: ______________________

5. Country of work
Open-ended question

______________________

6. Number of EGPA patients treated/managed in your Centre
Single-choice answer

- a) <10
- b) 11-30
- c) 31-50
- d) 50-100
- e) >100

## Macroarea 2: Diagnostic dilemmas

These questions must be applied in a scenario of a patient without a previous diagnosis of EGPA

7. Which represents the most important red flag EGPA for respiratory physicians? (This question is only for pulmonologist)
Single-choice answer

- a- A steroid-dependent asthma in absence of other explanatory comorbidities (e.g. adrenal insufficiency)
- b- Serum eosinophils > 1000 cell/mm3
- c- High rate of severe exacerbations of respiratory disease
- d- ANCA positivity
- e- Extra thoracic and nasal symptoms

8. Which represents the most important red flag  for EGPA for ENT specialists? (This question is only for ENT)
Single-choice answer

- a- Uncontrolled, adult-onset, non-allergic asthma
- b- Serum eosinophils > 1000 cell/mm3
- c- Severe recalcitrant CRSwNP
- d- ANCA positivity
- e- Extra thoracic and nasal symptoms

9. Which represents the most important red flag of EGPA for immunologist/rheumatologist? This question is only for rheumatologists, immunologists, specialists in internal medicine)

Single-choice answer

- a- Uncontrolled, adult-onset, non-allergic asthma
- b- Serum eosinophils > 1000 cell/mm3
- c- Pulmonary infiltrates
- d- ANCA positivity
- e- Systemic disease manifestations in a patient with eosinophilia

10. In which cases do you think it is more important to perform a biopsy (multiple answers allowed)?

Multiple-choice answer

- a- In case of suspected skin vasculitis
- b- In case of suspected glomerulonephritis
- c- In case of suspected nasal involvement
- d- In case of suspected peripheral nervous system involvement
- e- In case of suspected central nervous system involvement
- f- In case of suspected cardiac involvement
- g- In case of suspected gastrointestinal involvement

11. According to your clinical experience, how often a histological confirmation of granulomatous inflammation with eosinophilic infiltration is available in a patient with EGPA?

Single-choice answer

- a- < 10%
- b- 10-25%
- c- 25-50%
- d- >50%

12. According to your clinical experience, how often a histological confirmation of vasculitis is available in a patient with EGPA?

Single-choice answer

- a- < 10%
- b- 10-25%
- c- 25-50%
- d- > 50%

13. Do you usually recommend a regular follow-up with pulmonary function test?

Single-choice answer

- a- Yes, at least twice a year
- b- Yes, once a year
- c- Only in case of respiratory symptoms
- d- I don't usually recommend it

14. Do you consider the improvement of respiratory functional parameters (e.g. FEV1 or FVC) as a main outcome to be pursued in EGPA?

Single-choice answer

- a- Yes, in the majority of patients
- b- Yes, but only in patients reporting chronic respiratory symptoms
- c- No

15. In patients with a recent diagnosis of ANCA negative EGPA and no sign and symptom of cardiac involvement, which kind of cardiovascular assessment do you recommend?

Multiple-choice answer

- ☐ a- I ask for ECG and echocardiography.
- ☐ b- I refer the patient to a cardiologist.
- ☐ c- I ask for troponin
- ☐ d- I ask for cardiac MRI.
- ☐ e- I do not perform nor recommend any assessment for asymptomatic patients.

16. In patients with a recent diagnosis of ANCA negative EGPA and no sign and symptom of renal involvement, which kind of assessment do you recommend?

Multiple-choice answer

- ☐ a- I ask for routine urinalysis.
- ☐ b- I ask for serum creatinine, routine urinalysis and a protein to creatinine ratio.
- ☐ c- I refer the patient to a nephrologist.
- ☐ d- I do not perform nor recommend any assessment for asymptomatic patients.

17. In patients with a recent diagnosis of EGPA and suspected peripheral nervous system involvement, which is your preferred approach?

Mulltiple-choice answer

- ☐ a- I refer the patient to a neurologist.
- ☐ b- I ask for a nerve biopsy.
- ☐ c- I ask for EMG.
- ☐ d- I do not recommend any further assessment.

18. Which percentage of your patients is assessed together with other specialists?
Single-choice answer

- a) < 10%
- b) 10-25%
- c) 25-50%
- d) > 50%

**Macroarea 3: The challenge of treatment**

19. A complete remission (BVAS=0) with no OCS on maintenance can be achievable in
Single-choice answer

- a) All patients
- b) > 75% of patients
- c) 50% of patients
- d) < 50% of patients

20. How do you consider the goal of permanent discontinuation of maintenance OCS treatment?
Single-choice answer

- a) A primary outcome
- b) An important outcome, but contingent to the achievement of complete remission of disease
- c) A secondary outcome
- d) It is not one of my objectives in clinical practice.

21. How do you consider the goal of inhaled corticosteroids sparing?

Single-choice answer

- a) A primary outcome
- b) An important outcome, but contingent to the achievement of complete control of respiratory symptoms
- c) A secondary outcome
- d) It is not one of my objectives in clinical practice

22. In an EGPA patient treated with mepolizumab 100 mg/month plus OCS < 5 mg/die (prednisone equivalent) with a complete remission of disease, may you consider to upscale to mepolizumab 300 mg/month (in case reimbursement is arranged) in order to achieve a permanent discontinuation of OCS?

Single-choice answer

- a) Yes, in the majority of patients
- b) Yes, in selected patients
- c) Only in case of severe OCS-related side effects
- d) Never

23. In an EGPA patient treated with mepolizumab 300 mg/month and no OCS with BVAS 0 for more than 6 months, do you consider to downscale to mepolizumab 100 mg/ month?

Single-choice answer

- a) Yes, in the majority of patients
- b) Yes, in selected patients
- c) Only in case of mepolizumab-related side effects
- d) Never

24. In an EGPA patient, biologic naïve, with full clinical remission and persistent eosinophilia (>1000 cell/$mm^3$), which is your preferred approach?

Single-choice answer

- a) I suggest an increase in OCS.
- b) I suggest an increase in immunosuppressive treatment.
- c) I prescribe anti-IL5 agents
- d) I do not modify the treatment

25. In an EGPA patient, biologic naïve, with persistent clinical respiratory symptoms (ongoing steroid-dependent asthma with exacerbations) and no eosinophilia, which is your preferred approach?

Single-choice answer

- a) I suggest an increase in OCS/LABA.
- b) I suggest an increase in immunosuppressive treatment.
- c) I prescribe anti-IL5 agents.
- d) I do not modify the treatment.

26. For remission induction in severe EGPA, which is your preferred drug (males and non-potentially fertile women)?

Single-choice answer

- a) Cyclophosphamide
- b) Rituximab
- c) Mepolizumab alone
- d) Mepolizumab in combination with Cyclophosphamide
- e) Mepolizumab in combination with Rituximab
- Other: ____________________

27. For induction treatment in severe EGPA, I use:
Single-choice answer

- a) Combination treatment RTX + MEPO (i.e. I start MEPO within 3 months from the last infusion of RTX)
- b) Sequential treatment RTX + MEPO (i.e. I start MEPO at least 3 months after the last infusion of RTX)
- c) Both

28. For remission maintenance in patients treated with RTX at induction, how long do you treat your patients with RTX?
Single-choice answer

- a) I do not use RTX for maintenance
- b) 12-24 months
- c) 24-36 months
- d) > 36 months

29. In patients with a recent diagnosis of non-severe EGPA, what is you preferred approach for anti-IL5 agents?
Single-choice answer

- a) I prescribe anti-IL5 immediately at diagnosis.
- b) I prescribe anti-IL5 in relapsing patients.
- c) I prescribe anti-IL5 in case of failure of immunosuppressants.
- d) I prescribe anti-IL5 patients refractory to OCS

30. In patients with a recent diagnosis of severe EGPA, what is you preferred approach for anti-IL5 agents?
Single-choice answer

- a) I prescribe anti-IL5 immediately at diagnosis.
- b) I prescribe anti-IL5 in relapsing patients.
- c) I prescribe anti-IL5 in case of failure of immunosuppressants.
- d) I prescribe anti-IL5 patients refractory to OCS

31. When prescribing Mepolizumab, which is your preferred approach?

Single-choice answer

- a) I start with 100 mg/4 weeks
- b) I start with 300 mg/4 weeks
- c) I choose the dosage according to the severity of the symptoms
- d) I choose the dosage according to the localizations of disease
- e) I choose based on possible reimbursement

32. Which is, according to your judgement, a low dosage of OCS (expressed as prednisone equivalent)?

Single-choice answer

- a) < 10 mg/day
- b) < 7.5 mg/day
- c) < 5 mg/day
- d) < 2.5 mg>/day

33. How do you manage OCS and/or immunosuppressants dosage in a patient treated with anti-IL5 agents who has achieved a complete clinical response?

Single-choice answer

- a) I recommend a rapid tapering regime until the complete discontinuation of OCS and/or immunosuppressants.
- b) I recommend a tapering regimen until the complete discontinuation of OCS and/or immunosuppressants, to be achieved not before 12 months after anti-IL5 prescription
- c) I recommend a tapering regime of OCS and/or immunosuppressants, but I prefer to maintain a chronic low dosage of OCS and/or immunosuppressants in association with anti-IL5
- Other: ______________________

34. In your opinion, when would you prescribe benralizumab 30 mg/4 wks in a patient with a diagnosis of EGPA?

Multiple-choice answer

- [ ] a) With the same indications of mepolizumab 300 mg, as a first-line treatment
- [ ] b) As a second-line treatment, in case of suboptimal response to mepolizumab 300 mg in terms of clinical control of asthma.
- [ ] c) As a second-line treatment, in case of suboptimal response to mepolizumab 300 mg in terms of steroid-sparing.
- [ ] d) As a second-line treatment, in case of suboptimal response to mepolizumab 300 mg in terms of clinical control of EGPA (including non respiratory symptoms)
- [ ] Other: ______________________

## Macroarea 4: Patient-reported outcomes (PROMs)

35. On average, how often do you implement specific questionnaires for quality of life while visiting patients with EGPA?

Single-choice answer

- ( ) a) Never
- ( ) b) < 10%
- ( ) c) 10-50%
- ( ) d) > 50%

36. In your personal experience, what is the most valuable clinical goal reported by EGPA patients?

Single-choice answer

- ( ) a) Increase survival
- ( ) b) Improve quality of life
- ( ) c) Avoid steroid and/or immunosuppressant side effects
- ( ) d) Maintain the physical function and psychological balance

37. Which Questionnaire do you use to assess quality of life?

Multiple-choice answer

- [ ] a) Asthma Quality of Life Questionnaire (AQLQ) or miniAQLQ
- [ ] b) AAV-PRO
- [ ] c) Other (specify)
- [ ] d) None

38. Which questionnaire do you use in your clinical practice to assess asthma control?

Multiple-choice answer

- [ ] a) Asthma Control Test
- [ ] b) Asthma Control Questionnaire
- [ ] c) Asthma quality of life questionnaire
- [ ] d) None

39. Which questionnaire do you use in your clinical practice to assess ENT symptoms?

Multiple-choice answer

- [ ] a) Sino-Nasal Outcome Test 22
- [ ] b) VAS scale for different symptoms
- [ ] c) Sniffin sticks olfactory test
- [ ] d) None

## Macroarea 5: The role of patient advocacy organisations

40. Please select which are the most important activities of PAOs in EGPA according to your experience/opinion

Multiple-choice answer

- [ ] a) Educational and psychological support for the patients
- [ ] b) Referral of the patients to Centers with high expertise
- [ ] c) Connection with health system and political stakeholders
- [ ] d) Scientific activity and direct involvement in guidelines writing

41. What is your relationship with EGPA PAOs?
Single-choice answer

- a) I have no contact with any association
- b) I know some associations but I never spoke to them
- c) I'm in regular contact with a or many patients' associations but I never actively collaborated
- d) I'm in constant contact and actively collaborate with patients' association

42. Which is the percentage of patients with EGPA that has been referred to your Centre by a PAO?
Single-choice answer

- a) < 10%
- b) 10-50%
- c) > 50%
- d) > 75%

43. Which is the percentage of patients with EGPA specifically asking for information about PAOs?
Single-choice answer

- a) < 10%
- b) 10-50%
- c) > 50%
- d) > 75%

44. Does your hospital provide a dedicated space for PAOs?
Single-choice answer

- a) Yes and EGPA patients' associations are present in my hospital
- b) Yes but EGPA patients' associations are not present in my hospital
- c) no
- d) I don't know
