# Supplementary material for: Current management of eosinophilic granulomatosis with polyangiitis across Europe: insights from a multinational expert survey
Source: Rheumatology (Oxford). 2026 Apr 21;65(5):keag218. doi: 10.1093/rheumatology/keag218 (PMC13152655; doi:10.1093/rheumatology/keag218)
Supplement: keag218_Supplementary_Data [file keag218_supplementary_data.zip › rhe-25-3203-File003.docx]

**Supplementary material**

Supplementary Data S1. List of survey questions, subgrouped in 5 macroareas


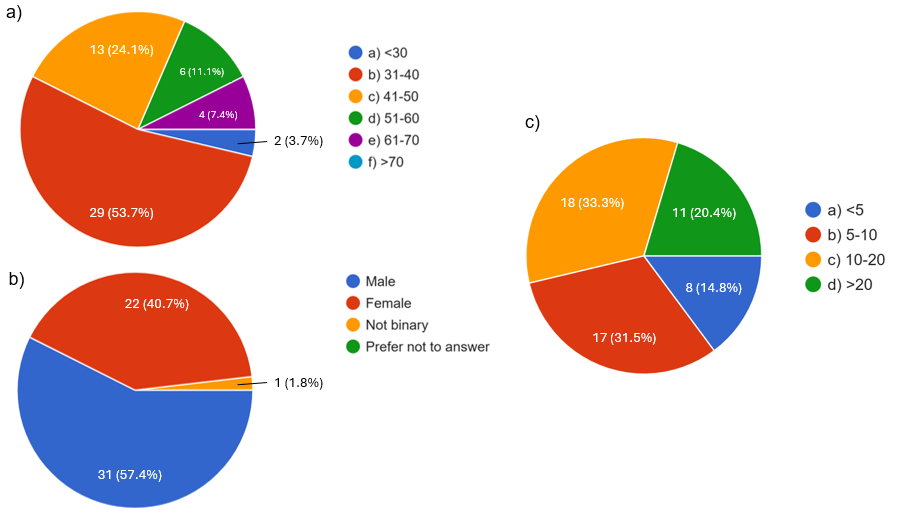


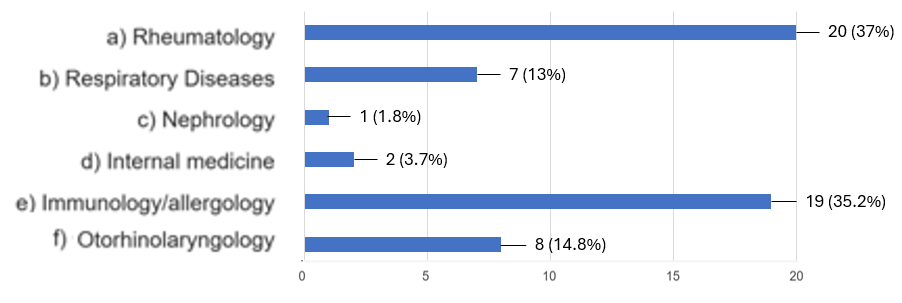


Supplementary Figure S1. Characteristics of survey participants population (n=54), stratified on age (a, expressed in years), sex (b), years of clinical practice in EGPA (c, expressed in years) and medical specialization (d; three participants declared two specializations)


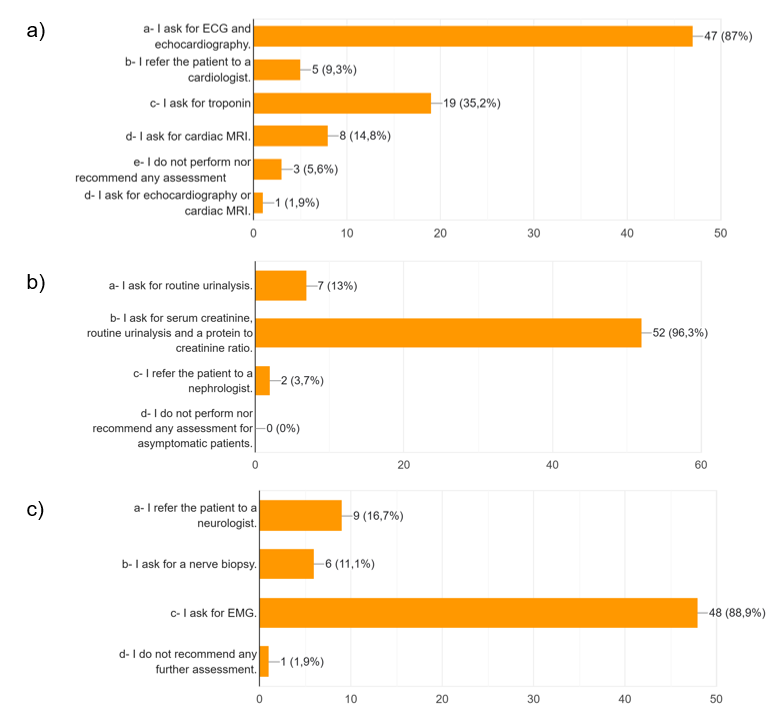


Supplementary Figure S2. Clinicians’ approach in case of suspected involvement of heart (a), kidney (b) and peripheral nerve (c) (multiple answers allowed).


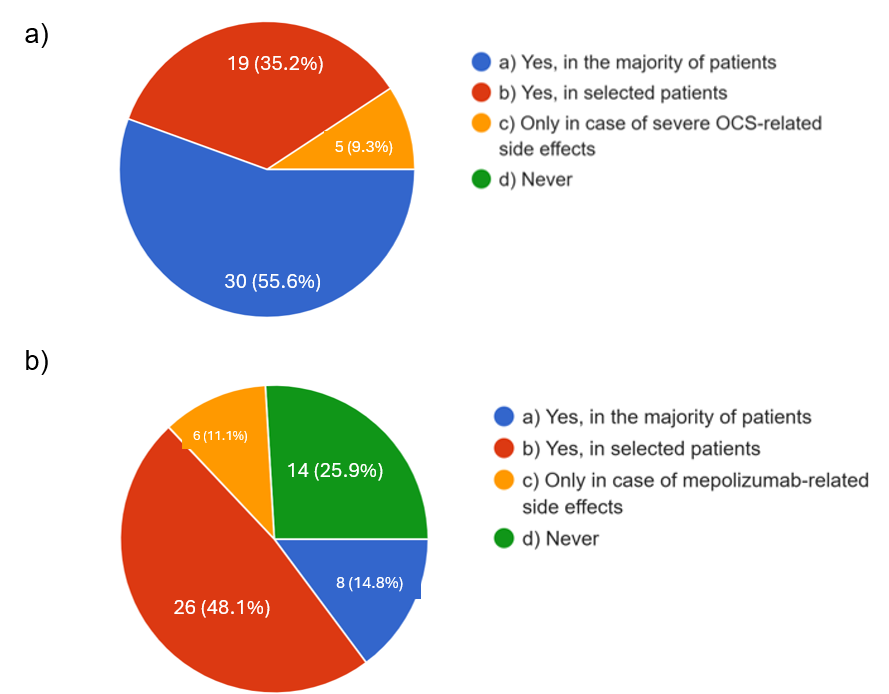


Supplementary Figure S3. Mepolizumab dosage during the maintenance treatment. a) Participants were asked about their perspective of upscaling mepolizumab from 100 to 300 mg/month to achieve a permanent discontinuation of OCS in patients with BVAS 0 taking daily low dose of OCS (prednisone < 5 mg). b) Participants were asked about their perspective of downscaling mepolizumab from 300 mg to 100 mg/month in patients with BVAS 0 and no daily OCS.


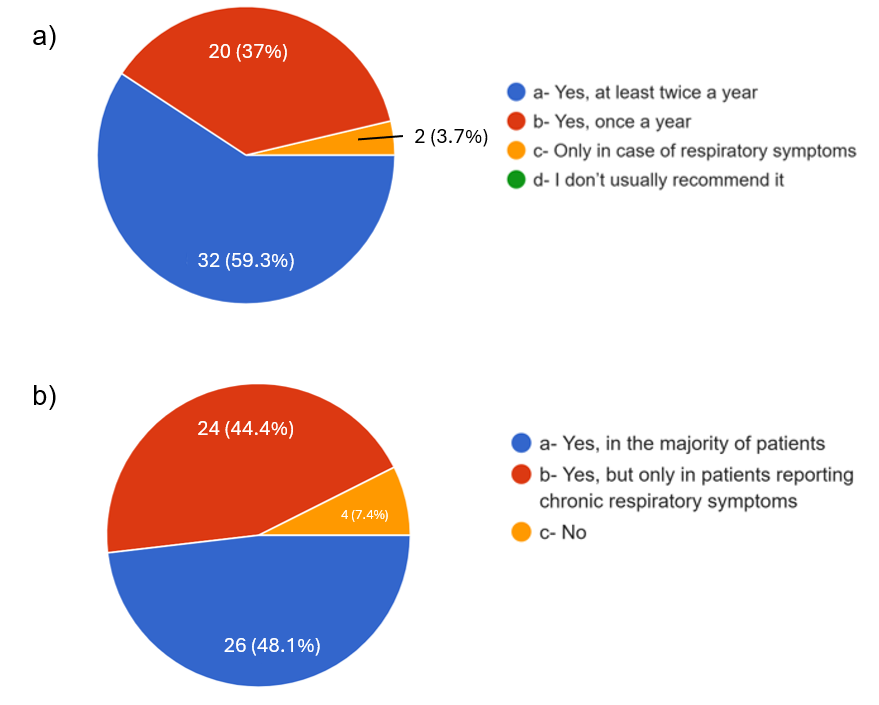


Supplementary Figure S4. a) Attitude of survey responders about the frequency of respiratory functional assessment usually prescribed in EGPA patients; b) Clinicians’ perspective about considering respiratory functional parameters (FVC and FEV1) as relevant outcomes to be pursued in EGPA management


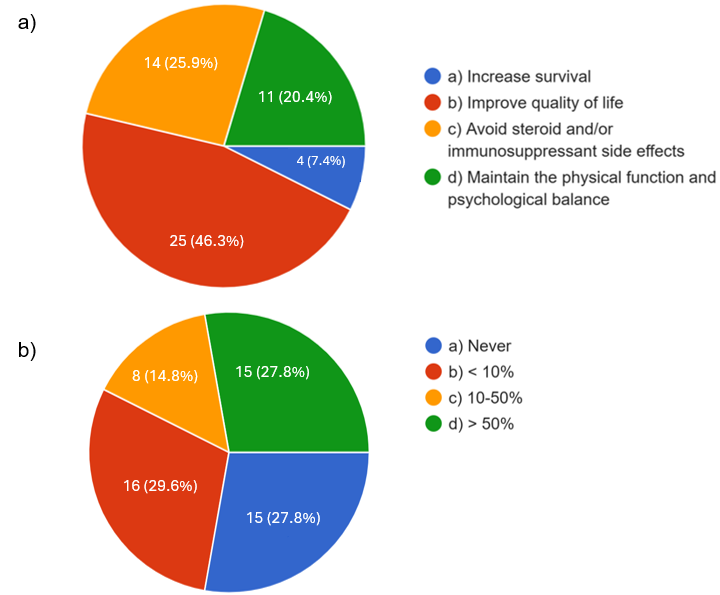


Supplementary Figure S5. Patients- reported clinical goals and implementation of PROMs in clinical practice. Survey participants were asked to report which is the most valuable goal reported by patients affected with EGPA (a) and to declare how often they implement PROMs (es. Validated questionnaires) while visiting patients with EGPA.
